# Ensemble Deep Learning-Based High-Precision Framework for Breast Cancer Detection from Histopathological Images

**DOI:** 10.3390/diagnostics16050653

**Published:** 2026-02-24

**Authors:** Faizan Ahmad, Arfan Jaffar, Ghazanfar Latif, Jaafar Alghazo, Sohail Masood Bhatti

**Affiliations:** 1 Department of Computer Science, Superior University, Lahore 547700, Pakistan; fizanciit@gmail.com (F.A.); arfan.jaffar@superior.edu.pk (A.J.); sohailmasood@superior.edu.pk (S.M.B.); 2 Department of Computing Science, Thompson Rivers University, Kamloops, BC V2C 0C8, Canada; 3 Math, Science and Technology Department, University of Minnesota Crookston, Crookston, MN 56716, USA; alghazo@crk.umn.edu

**Keywords:** breast cancer screening, convolutional neural network, ensemble learning, feature concatenation, overfitting mitigation, precision engineering, deep learning, histopathological images, vision transformers, cross-attention

## Abstract

**Background/Objectives**: Analysis of histopathological images is the absolute standard of breast cancer diagnosis. However, modern deep learning- and ViT-based architecture still struggle to capture effective local and global discriminatory patterns that tend to make architecture more complex, increasing the risk of overfitting and optimization problems. **Methods**: To address these problems, this paper proposes a four-phase hybrid framework that aims to enhance the feature fusion, improving the model’s strength, robustness, and generalization ability. In Phase 1, the BreakHis dataset was split patient-wise into a 70-15-15 manner to avoid data leakage, while extensive data augmentation, comprehensive normalization, and a five-fold cross-validation protocol were implemented to make the dataset more varied and reliably evaluated without bias. Phase 2 entailed the training of three CNNs (VGG16, ResNet50, and DenseNet121) and four ViTs (DeiT, CaiT, T2T-ViT, and Swin Transformer) independently to establish the strict baseline performance standards. In Phase 3, the CNN-based features were fused and classified with a soft voting mechanism to allow more stable and representative learning. Phase 4 depicts the Proposed Framework, which combines the two best-performing CNN and ViT models. Feature refinements were performed randomly by using Global Average Pooling and feature scaling, while a self-attention mechanism enabled the accurate cross-modal feature fusion. The generalization capability of the fused representation was further enhanced by the subsequent of dense layers followed by dropout. **Results**: XGBoost exhibited the highest performance among the evaluated ML classifiers, achieving 98.7% accuracy and 98.7% F1-score on BreakHis, while achieving 95.8% accuracy on external BACH dataset backed by Grad-CAM- and Grad-CAM++-based interpretability. **Conclusions**: By integrating CNNs and ViTs through self-attention, the proposed framework offers a robust and interpretable solution for automated breast cancer diagnosis.

## 1. Introduction

Breast cancer is a common malignancy among women and one of the biggest causes of cancer-related deaths in the world. There were approximately 2.3 million new cases and 685,000 deaths reported in 2020, with 317,000 new cases predicted to be diagnosed in the United States in 2025. Similarly, Asian and American women had experienced a rapid increase in both age groups (2.7% younger and 2.5% older) per year, exhibiting a negative impact on global health [1]. Tissue analysis under a microscope remains the gold-standard method of breast cancer diagnosis; currently, pathologists assess the type of tissue samples when handling the diagnostic process. However, manual assessment is time-consuming, subjective, and prone to inter-reader variability that underscores the need for a diagnostic workflow based on artificial intelligence (AI) [2]. Early computational techniques relied on classical machine learning (ML) classifiers, including k-nearest Neighbors (k-NNs), Decision Trees, and Support Vector Machines (SVMs), which are based on manually designed features such as texture, shape, and color. Altogether, these methods are beneficial because they can only process subtle, complex, hierarchical, and global structures in histopathological images [3,4].

Models like XGBoost, Multilayer Perceptron (MLP), and Random Forest have become very popular in recent years because they achieved high predictive performance, are highly interpretable, and are naturally robust to imbalanced datasets [2,4]. It is reported that fused features with deep learning and XGBoost achieved better classification results in medical image analysis. Similarly, we fed deep CNN features into XGBoost and achieved higher performance on the BreakHis dataset, but these models still faced number of challenges due to the complexity of spatial feature representations [3,5,6]. The introduction of deep learning has transformed the medical image classification like Convolutional Neural Networks (CNNs) such as VGG16, ResNet50, and DenseNet121 have been widely adopted because they can automatically learn hierarchical representations. CNNs are good at capturing local spatial patterns, but when trained on small datasets, they tend to overfit, have difficulty in modeling the long-range dependencies, and are critical for histopathological textures [5,7].

Vision Transformers (ViTs), which are self-attention-based models that leverage the long-range global feature relationships, have achieved palpable improvements over transformers [8]. The Swin Transformers (a hierarchical version of popular ViTs) have dominated classification on several medical imaging tasks, indicating that the Swin Transformer models are better than CNNs at learning the structural variations between tissues [9,10,11]. Nevertheless, ViTs have high data requirements and are computationally intensive; they also do not have the inductive biases of CNNs, such as locality or translation invariance [12]. CNNs are prone to overfitting on small or unbalanced datasets that negatively affect their performance and generalization ability [13,14,15]. ViTs, on the contrary, underfit or overfit due to tokenization granularity and often require pre-training on large datasets to achieve optimal performance [16]. CNNs perform well with regard to fine-grained local features, including tissue textures, cellular boundaries, and subtle morphological patterns, because they can only be trained to provide only localized spatial data. ViTs, on the other hand, are highly efficient at capturing the long-range dependencies and global contextual structure of the entire image. Hybrid CNN and ViT models achieved improved feature representations, higher classification performance, as well as greater sensitivity to image resolution, magnification, and staining regimes. Such synergy is particularly effective in histopathological image analysis, where the details of cells at the micro-scale level as well as the structure of tissues at the macro-scale level are important in providing a valid description of the disease. Thus, hybrid models could be useful for minimizing the inherent disequilibrium of using either global or local features to provide a globalized diagnosis. Ensemble and hybrid learning strategies have been widely researched to alleviate the shortcomings of individual models. CNN-based ensembles, namely those that employ soft voting schemes such as VGG16, ResNet50, and DenseNet121, have demonstrated dramatic improvements in predictive performance and resistance to predictive variability. Despite these advancements, the combination of state-of-the-art CNN and ViT models has received a little attention in the literature, suggesting that much greater performance improvements can be achieved through a more detailed integration [17,18]. Nevertheless, related work has barely discussed the hybrid use of the best-performing CNN and ViT models.

This paper presents a four-stage pipeline that integrates the strengths of CNNs, ViTs, and conventional ML models, such as XGBoost, Random Forest, and Multilayer Perceptron, to effectively classify breast cancer in histopathological images. During Phase 1, the BreakHis dataset, consisting of 7909 images, is resized, normalized, and augmented via stratified sampling in a 70/15/15 manner [3,17,19]. Phase 2 has baseline model evaluation in which three CNN-based models (VGG16, ResNet50, and DenseNet121) and four transformer-based classifiers (ViTs, Swin Transformer, etc.) are independently trained and evaluated to access their accuracy and overall performance. Other classical ML classifiers, such as Random Forests, Multilayer Perceptrons, and XGBoost, are used to classify the deep features [17,20,21]. Phase 3 includes the CNN ensemble model, in which all three CNN classifiers were assembled via soft voting, while the ensemble method is reported to achieve the higher accuracy [18,22]. Finally, Phase 4 combines the best-performing CNN (VGG16) and ViTs (Swin Transformer) at the feature level, while on the CNN and ViT outputs, Global Average Pooling (GAP) was applied independently to convert the spatial features to 1D, which could be easily integrated with the other model outputs, while the features were scaled to ensure all model outputs were on the same scale after GAP. The normalized features were fused using a Self-Attention mechanism to ensure the model is interpretable and to better leverage the strengths of each architecture. Dense layers followed by dropout were then added on top of fused and refined features to prevent overfitting in finite datasets and to maximize the feature discriminability. The final classification was performed using three Classifiers XGBoost, Random Forest, and Multilayer Perceptron (MLP), with XGBoost yielding the best accuracy and stability. The Proposed Framework achieved 98.7% accuracy, 98.6% precision, 98.7% recall, and 98.7% F1-score. The most innovative part of proposed approach is the design of a dense-attention fusion block, which includes the dense layers, dropout, and self-attention mechanisms that clearly focus on weighted CNN and ViT features. An ablation study clearly shows that adding fusion, attention, and dense components improves performance incrementally, indicating that the Proposed Framework is highly innovative, computationally efficient, and robust, ensuring reliable performance and offering a substantial contribution. Additionally, to ensure the robustness and generalization ability, the Proposed Framework was evaluated on an independent external BACH dataset, while Grad-CAM, Grad-CAM++, and global attention maps based on explainability analysis were implemented on breast cancer histopathological images to provide interpretable and clinically relevant insights into the model’s decision-making process.

**Figure 1:**
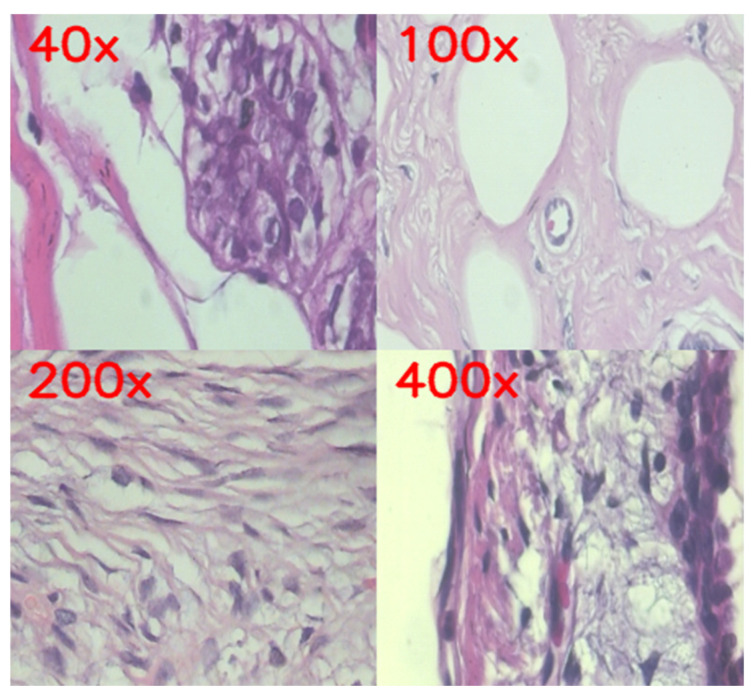
Histopathological images from BreakHis dataset with different magnification levels (40×, 100×, 200×, and 400×).

The Breast Cancer Histopathological Image dataset (BreakHis) is a popular benchmark utilized in automated diagnosis of breast cancer that consists of 7909 histopathological images gathered on 82 patients, which are divided into benign and malignant. The data is captured with images at four different magnifications, 40×, 100×, 200×, and 400×, which enables the study of tissue morphology in various magnifications, as shown in Figure 1 below. The global architectural pattern of the glandular structures and stroma can be identified at 40× and 100× magnification. When magnifications are increased to 200× and 400×, finer cellular features, such as nuclear size, chromatin distribution, and mitotic processes, can be observed, which are important for breast cancer diagnosis.

This multi-scale imaging approach is beneficial in training the models to learn both global and local features, as the deep learning architectures can learn deep features. CNNs that are good at extracting fine-grained local features, such as VGG16, can easily capture local texture and shape details, whereas Vision Transformers (ViTs) that are good at long-range dependencies, such as Swin Transformer, capture the long-range contextual relationships. The multifunctional features of these two classifiers, along with the Proposed Framework (CNN–ViT) built on them, were used to enhance the quality and predictability of automated breast cancer diagnosis.

The dataset has eight tumor types (four benign, i.e., Adenosis, Fibro Adenoma, Tubular Adenoma, Phyllodes Tumor) and four malignant (i.e., Ductal Carcinoma, Lobular Carcinoma, Papillary Carcinoma, and Mucinous Carcinoma). Samples are evenly distributed across four magnifications—40× (1995 samples), 100× (2081 samples), 200× (2013 samples), and 400× (1820 samples)—as shown in Table 1 below. This range allows models to yield both global tissue architecture and the cellular structure, which are specific to the BreakHis dataset.

## 2. Literature Review

Globally, breast cancer is the leading cause of cancer-related mortality, with an estimated 317,000 new cases predicted to be diagnosed in the United States in 2025. Similarly, Asian and American women had experienced a rapid increase in both age groups (2.7% younger and 2.5% older) per year, thus exhibiting a negative impact on global health [1]. Early diagnosis is vital in improving the patient’s prognosis, reducing the risk of complications, enhancing the patient’s survival, and aiding therapeutic interventions. Histopathological image analysis, which is regarded as the gold standard of breast cancer diagnosis, is traditionally conducted manually by pathologists as a result of scrupulous examination of tissue sections. Nevertheless, it is a time-consuming and laborious process that is likely to be affected by inter-observer bias or human error, especially due to fatigue. First-generation computer-aided diagnosis systems were mainly based on classical machine learning algorithms, such as k-nearest Neighbors (k-NNs), Support Vector Machines (SVMs), decision trees, and ensemble algorithms. These methods rely on handcrafted features, including manually extracted information on tissue textures, forms, and color patterns, as well as features derived from standard descriptors. Even these classical models have demonstrated strong potential. However, they are inherently constrained in their predictive ability by the quality, extent, and representational integrity of manually engineered features. Such types of handcrafted features do not always adequately reflect the morphological and structural heterogeneity typical in histopathological images, thereby limiting the models’ ability to be adaptive and to generalize across different tissue patterns and variations [3,7,11,23]. Although these models are effective for classification tasks, they do not generalize well to complex, high-dimensional histopathological images because they cannot capture the complex spatial patterns [3,23].

Ensemble models, which offer a strong regularization and interpretability including XGBoost, have also become a powerful alternatives for classification tasks in recent years [4,10,18]. Recent literature has shown that XGBoost performs better on medical image analysis, especially when deep features from Convolutional Neural Networks (CNNs) are used, and it has been effectively trained on CNN-generated features to achieve higher classification performance [1,4,24]. Deep learning and CNNs have completely transformed medical image analysis, with different architectures such as VGG16, ResNet50, and DenseNet121 demonstrating a strong performance in classifying breast cancer [25]. Such types of networks can learn hierarchical representations of features and spatial patterns that are essential for detecting the malignant structures in different tissue regions [7,8,26]. VGG16 is a deep network with sixteen weight layers and a fixed kernel size, allowing for a more detailed extraction of local patterns [27,28]. ResNet50 uses the skip connections, which reduce the vanishing gradient problem and enable the training of significantly deeper networks [26,29]. CNN models can overfit even with good performance on relatively small datasets like the BreakHis, especially when the dataset is not artificially augmented or augmented after the train–test split. Further, CNNs have an intrinsically local receptive field, limiting their ability to represent global contextual information (which can be very important for global tissue characterization) [7,8].

Building on the achievements of transformers in natural language processing, Vision Transformers (ViTs) have found more applications in medical image analysis. ViTs can capture long-range relationships via self-attention mechanisms and extract global contextual information from histopathological images. Despite these benefits, traditional Vision Transformers lack the inherent inductive biases of Convolutional Neural Networks, such as locality and translation invariance, and typically perform best with large datasets and substantial computational resources [3,8,12]. Swin Transformer, a variant of ViT, addresses many of ViTs shortcomings, such as overfitting and redundant features representation. The hierarchical structure is sufficient to capture both local and global contextual patterns, leading to higher and more expressive feature representations [12,30]. Hybrid models have already proven better performance than traditional CNNs in analyzing breast cancer from histopathology images. Specifically, the Multi-View Swin Transformer (MSMV-Swin) that leverages multiple perspectives to capture the richer features is more powerful and can represent features with higher performance due to its generalization across different tissue structures and magnifications [10,13,16]. However, Vision Transformers are resource-intensive, required very large datasets to perform optimally, and can become impractical in resource-constrained environments when datasets are limited or hard to acquire [11,31,32]. Recent research has focused on the strengths of hybrid architectures by combining the Convolutional Neural Networks (CNNs) with variants of Vision Transformers (ViTs) for medical image classification. This method achieved high-quality diagnostic results, with lower diagnostic errors than single CNN or ViT models due to combination of local CNNs with global ViT representations. These results highlights the importance of hybrid frameworks for capturing the multi-scale and multilevel features, especially in complex medical imaging problems where both small-scale features and large-scale context are equally important for accurate classification [16,18,33]. The CNN extracted the fine-grained local features, while ViT captured the long-range contextual dependencies, with a fused embedding used for final classification [34,35]. Evaluation metrics, such as accuracy, precision, recall, and F1-score, showed that the hybrid model achieved excellent results across all datasets, with reported accuracies of 99.62% in DDSM and 100% in MIAS. These findings not only indicate that the model has strong generalization potential but also demonstrate the benefits of combining local and global feature representations. The results also indicate that the hybrid CNN–ViT-based models can significantly improve diagnostic performance with low error, underscoring their potential for clinical breast cancer diagnosis [36,37].

Overfitting in deep learning models is a very critical problem due to scarcity of data that is prevalent in the medical imaging sector. Overfitting is commonly overcome using a number of traditional methods. For example, during training, dropout randomly blocks some neurons that cannot co-adapt, leading to the learning of higher-quality features. In batch normalization, inputs are normalized at each layer, stabilizing training by enabling faster convergence and reducing sensitivity to initialization. Data augmentation is an artificial method that enlarges the training set by introducing variations in the training data, such as rotation, scaling, and flipping, to allow the classifier to learn more versatile representations. All these methods improve the generalization and model performance, yielding more reliable findings on unseen medical images [33]. However, Vision Transformer (ViT) underfitting can be caused by coarse-grained patch tokenization, whereas fine-tuning on a small dataset can lead to overfitting. This is because ViT’s weak architectural inductive biases make them highly reliant on large training datasets and various strategies to ensure the successful and stable training [12,29,38,39,40]. Hybrid learning algorithms have been extensively used to overcome the drawbacks of a single model. Specifically, robustness and predictive performance can be improved using ensemble of Convolutional Neural Networks (CNNs) based on soft voting; for example, soft voting across an ensemble of VGG16, ResNet50, and DenseNet121 has been shown to outperform each individual architecture for breast cancer histopathology classification and to leverage the strengths of each architecture to complement the others [2,3,4,16].

Recent studies in 2023–2025 have made significant strides in histopathology image analysis by embracing a weakly supervised learning paradigm and transformer models. Transformers integrated with multiple instance learning (MIL) have gained popularity to model global contextual relationships in both whole-slide and patch-based histopathology images [41]. Attention mechanisms are widely used in transformer-based MIL frameworks to identify discriminative regions, thereby enhancing the classification robustness without requiring the pixel-level annotations [10,42,43]. Meanwhile, transformer-only architectures and self-supervised learning methods have emerged as highly effective in learning powerful representations and improving generalization across histopathological datasets [43,44]. Furthermore, very recently, foundation model-based pipelines and large-scale pre-trained Vision Transformers have been explored for cancer sub-typing and validation across datasets, showing promising results in computational pathology [41,42,43,44].

Despite these advancements, many such approaches are either bound to complex MIL pipelines or large-scale transformer models. In the present work, on the other hand, a lightweight and selective CNN–ViT fusion strategy is pursued, in which only the top-performing backbones are fused using a dense-attention module and the effectiveness of each component is systematically evaluated through ablation [21,40]. Advanced fusion schemes have also been considered in recent work, while the proposed Token Mixer is a transformer-based architecture tailored for histopathological tokenization. Intra-class discrimination has been further promoted in invasive ductal carcinoma detection in supervised learning techniques, like SupCon–ViT. Confusion matrices, ROC curves, Grad-CAM, global attention maps, and feature importance visualizations are some of the techniques that can help the clinicians understand how models make decisions and build confidence in AI systems. Performance measurement tools such as area under the curve (AUC), precision, recall, F1-score, and standard deviation are crucial for evaluating the performance, especially when the dataset is class-imbalanced [10,14,16,28,33].

Literature reviews from 2020 to 2025 highlight the various limitations of case-based breast cancer histopathology classification, as summarized in Table 2. The main limitations are a high reliance on the training dataset, complex computations, and poor interpretability. Models that are trained on particular datasets can be very difficult to generalize and can be too complicated to implement in clinical practice in real time. The small size of available datasets can lead to overfitting, and it is impossible to train all models on large-scale datasets, which is rather expensive.

The main approaches to overcoming those shortcomings are data augmentation, transfer learning, model pruning, and adopting more efficient architectures. Overfitting can be alleviated through regularization, visualization, and explainable AI techniques. The next round of research should aim to optimize the models, making them perform well on small datasets with lower computational demands, enhancing their clinical usefulness and practical implementations.

## 3. Methodology

### 3.1. Phase 1: Data Preparation

In the Proposed Framework, two publicly available datasets, BreakHis and BACH, were utilized. BreakHis dataset includes 7909 histopathological high-quality and well-marked slides from 82 patients, acquired at four magnification levels (40×, 100×, 200×, 400×). All images are stained with H&E and marked as benign and malignant, with sub-type information. However, precise demographic data, age, sex, or other patient-specific information are not released to the public. The dataset adheres to the typical inclusion criteria, namely confirmed breast tumor diagnosis and good-quality histopathological slides, but the original creators eliminated the slides that have strong artifacts, poor stains, and low resolution to guarantee effective model training.

**Figure 2:**
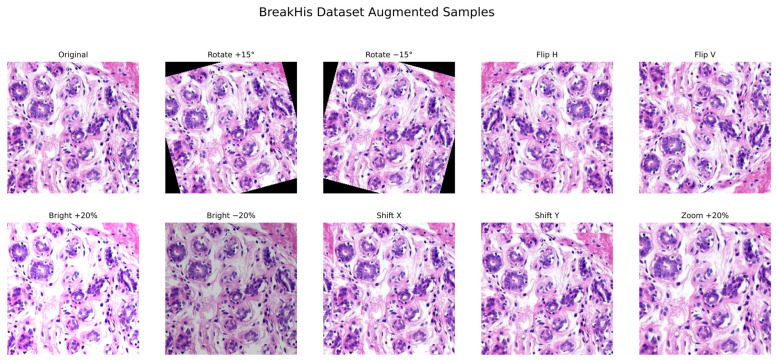
BreakHis histopathology sample images with applied augmentation (flip, rotation, shift, zoom, brightness).

To avoid data leakage, the dataset was split patient-wise into training, validation, and test sets in a 70-15-15 ratio. Images were downsized to 224 × 224 × 3 for input to the model and normalized to the 0–1 pixel range. On-the-fly data augmentation was implemented on the training set with ImageDataGenerator using TensorFlow by random rotations (both clockwise/anticlockwise, ±90°), horizontal and vertical flips, zooming, shifts, and changes in brightness/contrast, applied after patient-wise splitting as shown in Figure 2. Additionally, 5-fold cross-validation was utilized on the training set to ensure model robustness and consistency. The preprocessing and augmentation pipeline illustrated in Table 3 below.

The BACH dataset comprises 400 high-resolution H&E-stained breast histology samples, grouped into four classes: normal, benign, carcinoma, and invasive carcinoma. In the current study, classes were split into benign (normal + benign) and malignant (carcinoma + invasive carcinoma), and the dataset was used exclusively for external testing while preprocessing and data cleaning were also performed to make the dataset compatible with the trained model. The overall goal of applying BACH was to compare the generalization performance and the strengths of the Proposed Framework on external, unseen data.

### 3.2. Phase 2: Evaluation of Baseline Models

The second phase involved implementing of seven deep learning architectures as baseline models. The architectures were trained on the BreakHis train set, tested on the BreakHis test split, and evaluated on the external BACH dataset. Three CNNs (VGG16, ResNet50, DenseNet121) and four ViT-based variants (Swin Transformer, DeiT, CaiT, T2T-ViT) were trained independently to compare the performance on a fair basis. In the CNN models, the deep features of VGG16, ResNet50, and DenseNet121 were directly fed into fully connected layers followed by a softmax. In the case of transformer models, the images were broken down into fixed-size patches, flattened using patch tokenization, and 1D token vectors were linearly projected to the images as mathematically shown in Equations (1) and (2), which allowed transformers to be used to process the images.

(1) $$Patch\;Tokenization=x\in R^{H\times W\times C}\;\to \{p_{1},p_{2},\ldots p_{n}\}$$

(2) $$T_{i}=E.P_{i}+b$$

Patch embedding is infused with its specific positional encoding, and the result is the first sequence representation, denoted as $T_{i}$, that is described by Equation (3), while the learnable classification token [CLS] is placed at the start of the sequence, as shown in Equation (4).

(3) $$z_{0}=[T_{1}+P_{1},T_{2}+P_{2},\ldots \ldots T_{m}+P_{n}]$$

(4) $$Z_{0}=[{CLS,\;T}_{1}+P_{1},T_{2}+P_{2},\ldots \ldots T_{m}+P_{n}]$$

The sequence is subsequently run through multiple encoding layers by the transformer. Two operations were performed in each layer: multi-head self-attention (MSA) and a feed-forward network (MLP). In Equation (5), the transformer encoder has two sub-layers, the second of which is defined by Equation (6). Lastly, the output from of the previous encoder layer for the [CLS] token is sent through a classifier head for an earlier prediction, as defined in Equation (7).

(5) $$Z_{1}=MSA(LN(Z_{i-1})){\;+\;Z}_{l-1}$$

(6) $$Z_{n}=MLP(LN(Z_{l})){\;+\;Z}_{l}$$

(7) $$y=Softmax(Z_{Cls}\;.Z_{l}+b_{Cls})$$

where x is the input image, H is the height, W is the width, and C is the number of channels (e.g., RGB 3), (p1,p2,p3…pn) are the extracted patches from image x, E. a is the learnable embedding matrix, Pi is the patch, b is the learnable bias, $T_{i}$ is the token embedding, and $P_{i}$ is the embedding of each patch, while model capabilities are measured using key metrics such as accuracy, precision, recall, F1-score, and AUC-ROC.

### 3.3. Phase 3: CNN Ensemble via Soft Voting

VGG16 was chosen for its simplicity, popularity, and efficiency at capturing basic visual features, but it has less capacity to learn more complex and abstract patterns. To mitigate this, ResNet50 was added, whose residual connections enable the model to learn far deeper representations without degradation problems associated with vanishing gradients. To further enhance feature learning, DenseNet121 was implemented, which connects each layer to all its prior layers, thereby encouraging feature reuse and effective gradient flow. Despite the benefits of each model, using a single architecture can result in biased or sub-optimal outcomes, which is why the ensemble learning approach was used. The three CNN models, VGG16, ResNet50, and DenseNet121, were used to obtain a feature combination through a soft voting procedure as mathematically shown in Equation (8). The final class-wise probability vector is computed as elementwise, and the average of the three probability vectors is $P_{\left(final\right)}$. Once the averaged probability vector $P_{\left(final\right)}$ is obtained, the final predicted class y is selected by taking the index of the maximum value in that vector. In other words, the class with the highest confidence score after ensemble averaging is considered the final output, as shown in Equation (9).

(8) $$\begin{array}{cc} P_{final}= & 1/3(Softmax(VGG16(x))+Softmax(ResNet50(x))\\ & +Softmax(DenseNet121(x)) \end{array}$$

(9) $$y=arg\_max(P_{final})$$

### 3.4. Phase 4: Proposed Framework Construction

The Proposed Framework is based on the selected top CNN and top ViT, extracting features by initializing both with pre-trained ImageNet weights, with input images resized to 224 × 224 × 3. In case of the top CNN, the convolutional base was frozen, and only the final dense layers were fine-tuned on the top ViT. The first few blocks of the transformer (top ViT) were frozen while the final few blocks and the final classification head were fine-tuned only on the target dataset. After feature extraction, Global Average Pooling (GAP) was used to reduce spatial dimensions while normalization was applied to standardize the embedding scales. A self-attention mechanism was then added to combine the features of both models, with the most illuminating ones being highlighted. To allow further dimensionality reduction, improve pattern discrimination, and to prevent overfitting, dense layers followed by dropout were added to the refined and fused vector. Lastly, they were classified using XGBoost, while the additional experiments were conducted with MLP and RF. Early stopping (patience = 10), L2 regularization, and learning rate scheduling were used to improve training stability. Equations (10) and (11) described mathematically the Global Average Pooling (GAP) process to summarize each feature map into a single representative value and reduce the spatial dimensions. Equation (12) highlights the normalization process that standardizes the pooled feature vectors to a common scale, enhances numerical stability, ensures consistent inputs to the fusion, and classification stages.

(10) $$Global\;Average\;Pooling=\frac{1}{H\times W}\sum_{i=1}^{H}\times \sum_{j=1}^{W}x_{ijc}$$

(11) $$Z=[z_{1},z_{1}\ldots \ldots z_{n}]\forall z_{i}\in {\to R}^{d}$$

(12) $$X_{norm}=\frac{x-x_{min}}{x_{max}-x_{min}}\;$$

H is the height, W is the width, $x_{ij}$ represents the value of feature at position (i,j), c represents the channel, and the result will be in 1D vector. Whereas in Equation (11), $z_{i}$ is the token embedding excluding CLS, n is the number of patches, and the resultant vector will be 1D. This operation takes the multidimensional features of the maps and converts them into a compact, one-dimensional feature vector.

In Equation (12), x is original value, $x_{min}$, $x_{max}$ are the minimum and maximum value of the feature vector, and $x_{norm}$ normalized the values to 0–1. Rather than simply concatenating or averaging features, an attention mechanism was used to identify the most important features from CNN and ViT, enabling the model to focus on meaningful parts, as defined in Equations (13) and (14) below.

(13) $$Z_{f}=[Z_{CNN}][Z_{ViT}]$$

(14) Self_Attetnion(Zf)=Softmax[(zQzfd×WKzf)^T×(WKzf)

(15) $$y=W_{X}+b$$

(16) $$y^{-}=r\;⊙y,\;where\;r_{1}∼\;Bernoulli(p)$$

where $z_{f}$ = [$z_{ViT}$][$z_{CNN}$] are the concatenated features of vector, $W_{Q}{,W}_{K},W_{V}$ are the learnable weights, d shows the key of dimensionally vectors, x is the input vector (Attention output), W are the matrix weights, b is bias, and y is output vector. In Equations (15) and (16), y is the input activation, r is binary mask vector, p is probability of keeping neurons active, ⊙ is elementwise multiplication, and $\tilde{y}$ is the output after applying dropout. Finally, three highly dissimilar and powerful machine learning algorithms were selected for the classification task: XGBoost, Random Forest, and Multilayer Perceptron (MLP). The reason for choosing these three ML classifiers is their unique and diverse strengths, as well as their powerful and comprehensive performance comparison.

### 3.5. Experimental Setup and Implementation Details

All experiments were conducted with fixed hardware and software settings to ensure fair and reproducible results, as shown in Table 4 below. The Proposed Framework was trained on the Adam optimizer on TensorFlow, while the learning rate was set to 1 × 10^−4^ and decreased dynamically by 0.1 per validation step. The model was trained using categorical cross-entropy loss for 70 epochs with a batch size of 32. To enhance generalization and to avoid overfitting, random rotations, horizontal/vertical flipping, and data zooming were employed. Moreover, early stopping was used to avert unnecessary training once it converged and testing was performed on NVIDIA RTX 3090 card with 128 GB of system RAM. Moreover, the details of the experimental setup and model weights were derived from pre-trained ImageNet parameters in the final Proposed Framework. As a result, Grad-CAM, Grad-CAM ++, and global attention maps were used to visualize the discriminative parts and improve the comprehensibility of the diagnostic process.

**Figure 3:**
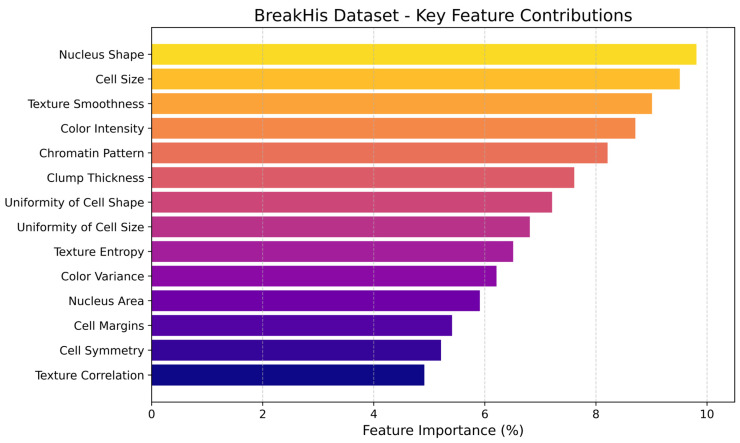
Key feature contributions and feature importance scores of BreakHis dataset in the Proposed Framework.

Enhanced feature importance visually depicts the relative contributions of different morphological, textural, and color-based features of breast cancer classification using the BreakHis dataset, as shown in Figure 3 below. The aim was to identify which histopathological image features are most influential in distinguishing between benign and malignant cases.

## 4. Experimental Results

### 4.1. Evolution Matrices

The Proposed Framework was evaluated on two histopathological datasets, BreakHis and BACH, while the number of metrics was calculated to assess robustness and generalization performance, including precision, recall, F1-score, AUC, and standard deviation (STD).

**Figure 4:**
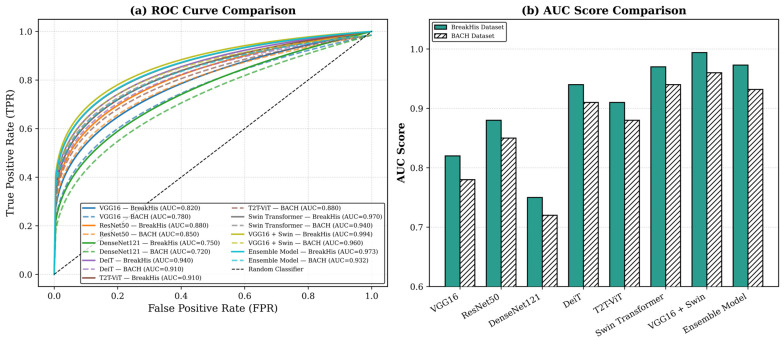
ROC curves and AUC scores for multiple models on the BreakHis and BACH datasets. (**a**) ROC curve comparison. (**b**) AUC score comparison.

As illustrated in the curve and bar chart in Figure 4 below, part (a) illustrates the comparison to show the trade-off of TPR versus FPR of all the competing models. The Proposed Framework had the highest AUC and good generalization ability without overfitting across all models on both datasets, with part (b) showing the AUC score comparison of every model. CNN-based models, i.e., VGG16 and ResNet50, have relatively low AUC values as compared to transformer-based models, while BreakHis and BACH had the highest AUCs of 0.994 and 0.960, respectively. This highlights the complementary nature of CNN- and Transformer-based architectures as the CNN detects fine-grained local features, whereas the Swin Transformer focuses on global contextual patterns. The consistency in the Proposed Framework across two datasets further establishes that it has a good balance between bias and variance, ensuring that it does not overfit and can be generalized to other heterogeneous histopathological image distributions.

**Figure 5:**
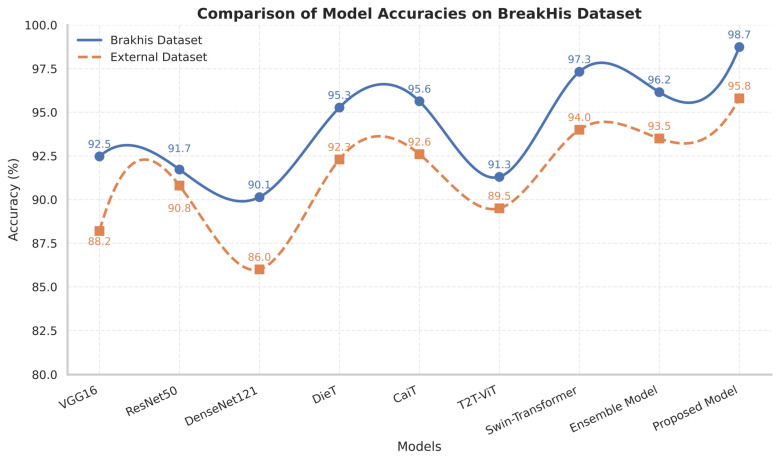
Comparative analysis of model performance based on accuracy (%), evaluated on the internal BreakHis and external BACH datasets.

The major concern of comparative performance analysis was to evaluate the generalization potential of the Proposed Framework in both microscopic and clinically relevant settings. The BreakHis dataset was chosen for training and testing because the number of breast tissue samples at various magnifications (40×, 100×, 200×, 400×) are large, optimal for feature extraction, and possess learning representations with deep architectures. Thus, validation was mainly applied to the BACH dataset, which comprises the annotated images of breast histology. The final accuracy plot across the BreakHis and BACH datasets are compared for nine deep architectures (VGG16, ResNet50, DenseNet121, DeiT, CaiT, T2T-ViT, Swin Transformer, Ensemble Model, and the Proposed Framework), as shown in Figure 5 below. The highest accuracy of the Proposed Framework on the BreakHis dataset was 98.74%, and the validation accuracy on the BACH dataset was 95.80%. Conversely, the Proposed Framework performed consistently across the BreakHis dataset, demonstrating that it could learn discriminative patterns from breast histopathological images and has the strong ability to generalize them.

**Figure 6:**
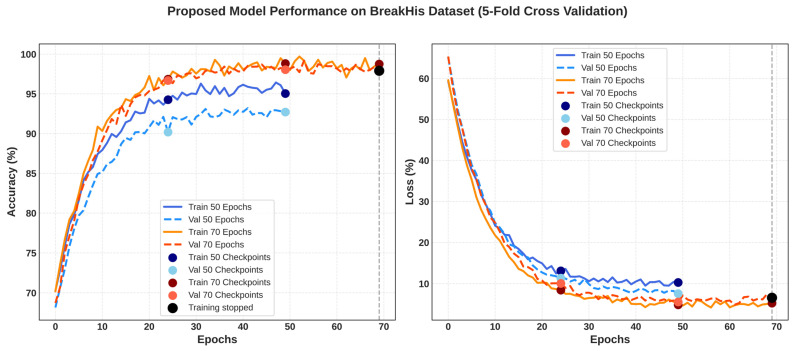
Training, Validation Accuracy and Training, Validation Loss of the Proposed Framework on the BreakHis dataset over different epochs with checkpoints.

The effectiveness of the Proposed Framework on the BreakHis dataset is demonstrated by comparative learning behavior reflected in Figure 6 below. The BreakHis dataset results show the mean training and validation performance across 5-fold cross-validation, and it was primarily used to train and internally validate the model.

The validation accuracy is slightly lower than the training accuracy at 50 epochs, indicating a small generalization gap, but training and validation curves converge after 70 epochs, while the validation loss stabilizes, indicating that the model continues to perform well. Scatter points highlight chosen checkpoints, while black markers and dashed lines identify the training stop point where early stopping was used. In general, the curves show that the Proposed Framework performs extremely well, with low loss across all folds, resulting in strong generalization and good resistance to overfitting.

**Figure 7:**
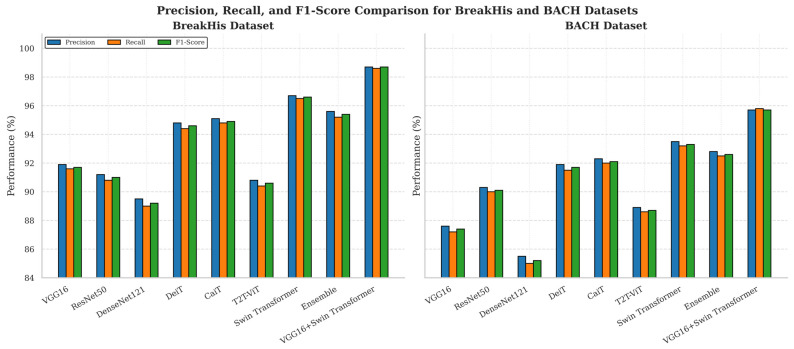
Comparison of Precision, Recall, and F1-Score for multiple models on the BreakHis (**left**) and BACH (**right**) datasets. Each subplot shows the performance of the same models across the three metrics.

The analysis of comparative performance across nine deep learning architectures is shown in Figure 7 below on both BreakHis and BACH breast histopathology datasets. Each model was trained on the BreakHis dataset across 5-fold cross-validation where each fold served as a validation split, while the remaining folds served as the training set. The cross-validation approach reduces the bias and variance, thereby improving the consistency of the reported measures. All models were subsequently evaluated using the BreakHis validation folds and assessed on external BACH dataset to examine their generalization across various data sources and imaging condition.

Three standard classification measures such as precision, recall, and F1-score were used to measure the performance of the Proposed Framework. These indicators highlight a moderate view of the model’s performance in accurately identifying cancerous tissue and reducing the false positives. The Proposed Framework performed better on both datasets, having 98.7% precision, 98.6% recall, and 98.7% F1-score on BreakHis, while having 95.7%, 95.8%, and 95.7% on the external BACH dataset, respectively.

Interestingly, the slight and steady decrease in performance is approximately 3% between internal BreakHis validation and external BACH testing, indicating that the Proposed Framework does not overfit the training set and learns the domain-invariant morphological representations that are applicable to other histopathology slides and stain variations.

### 4.2. CNN–ViT Feature Fusion Classification Results

An ablation study is conducted to examine the contribution of each proposed part (Fusion, Attention, Dense) as they are gradually introduced, beginning with the simplest feature fusion. All additions enhanced the classification performance, demonstrating that attention improves the discriminative feature selection, whereas the dense layers increase feature compactness and reduces redundancy. Deep learning models are still very promising for medical image classification, but it can only be realized when the features extracted from images are generalized across different datasets. The Proposed Framework combines the strengths of Convolutional Neural Networks (CNNs) and Vision Transformers (ViTs) to provide stronger feature representations and more complementary feature space.

Among the best-performing CNN and ViT variants, most stable and refined features were selected. An attention mechanism was used to extract, normalize, and channel-fuse features to learn channel-wise weights to adapt the two sets of features. This methodology allowed the model to focus on the most significant discriminative features in one set while ignoring the weak features in others. To overcome overfitting, the fine-fused features were used as inputs to the classifiers such as Random Forest (RF), XGBoost, and Multilayer Perceptron (MLP) to evaluate their performance.

The BreakHis dataset was tested across five-fold cross-validation to provide a fair and trustworthy performance comparison, as depicted in Table 5 below. Both classifiers demonstrated excellent performance when integrating the attention mechanism with a densely connected layers across all evaluation metrics.

The attention mechanism significantly enhanced precision and F1-score, even when fused on its own, demonstrating a consistent performance. Moreover, the dense layers helped to enhance performance by minimizing noise and reducing feature dimensionality. XGBoost demonstrated the best results among all classifiers, with 98.74 ± 0.14%, 98.70 ± 0.18%, accuracy, and F1-sore, respectively.

To assess generalization, the Proposed Framework is evaluated on the BACH dataset, which was not cross-validated and used entirely as an external validation set, while performance trend on BACH was quite similar to the BreakHis, as shown in Table 6 below. The application of attention mechanisms and dense layers consistently improved the performance of all classifiers. Once again, the XGBoost classifier achieved the best results on the validation set, with 95.8% and 95.75% accuracy and F1-score, respectively, indicative of its strong resilience on the unknown dataset. MLP and RF classifiers were once again strong, with accuracy of 94.8% and 94.2%, respectively. Despite the minor decrease in the performance on BACH because of variations in domains, these findings confirm that the Proposed Framework enables strong cross-dataset generalization.

XGBoost was the most stable across two datasets, which can be attributed to its gradient-boosting classifier, that effectively captured the nonlinear interactions and subtle differences in the merged feature space. It demonstrates a strong generalization, as evidenced by its consistent performance on the external BACH dataset as compared to the MLP and RF. In sum, the experimental results demonstrate that the attention-directed CNN–ViT fusion with dense-layer refinement produces a strong and noise-resistant representation that enables the high-performance classification, as summarized in Table 7.

### 4.3. Visual Explainability Analysis Using Grad-CAM and Global Attention

The visual interpretability findings of all observed CNNs and ViTs, as well as the Proposed Framework, on the BreakHis dataset are shown below. We used Attention maps, Grad-CAM, and Grad-CAM++ methods to understand the behavior of various CNN and ViT variants, where Grad-CAM annotates the image features that contribute most strongly to the model prediction by using gradients from the target concepts directed to the last convolutional layers. An extension called Grad-CAM++ produces the sharper and more localized heatmaps, particularly when several regions contribute to a class. Attention maps were also employed with ViT variations, where attention models rely on self-attention patterns to locate areas of interest while attention maps indicate the transformer’s focus during inference. This visualization offers a complementary insight, like Grad-CAM and Grad-CAM++ highlighting the class-discriminative regions, while the attention maps indicate the model’s focus without direct supervision.

**Figure 8:**
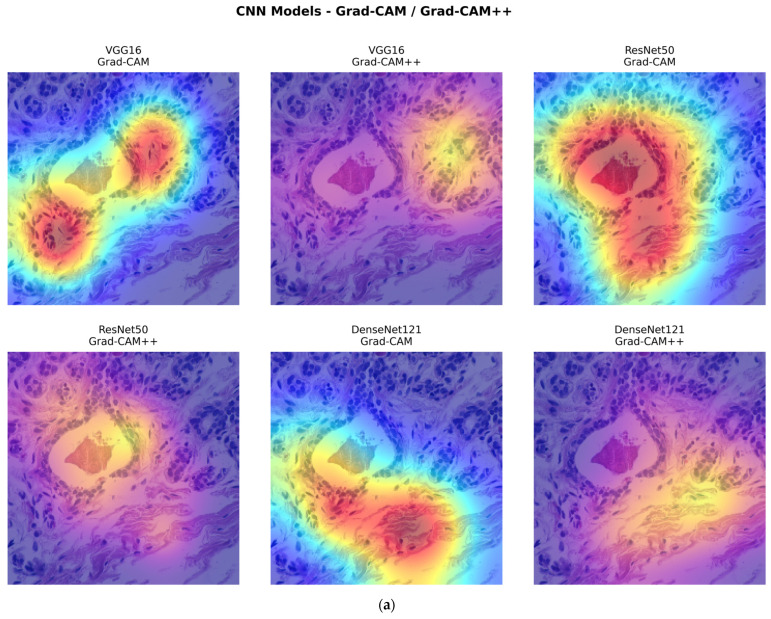

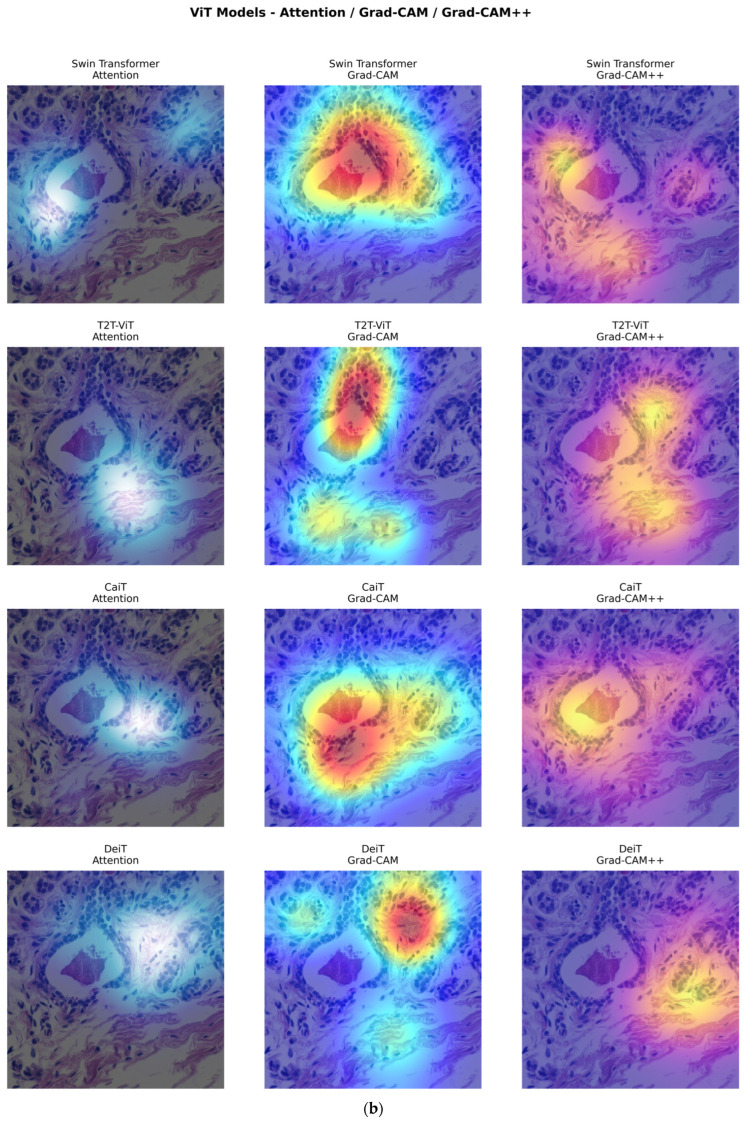

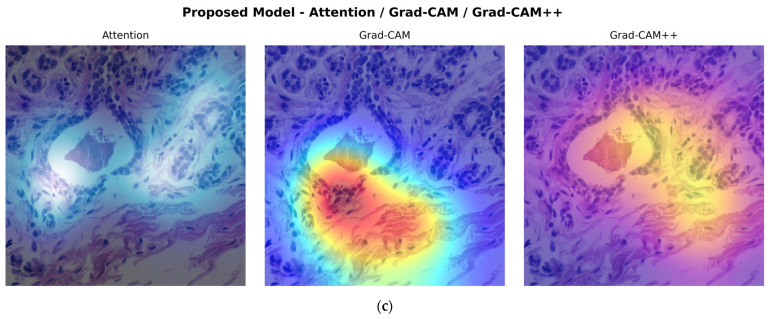
(**a**) Visualization of CNN variants using Grad-CAM and Grad-CAM++ on BreakHis histopathology images. (**b**) Visualization of Vision Transformer variants using Grad-CAM, Grad-CAM++, and Attention maps on BreakHis histopathology images. (**c**) Visualization of the Proposed Framework, Swin Transformer attention map, and the fused VGG16+Swin Transformer model using Grad-CAM and Grad-CAM++ heatmaps.

Figure 8a below depicts the CNN-based variants with Grad-CAM and Grad-CAM++, while Figure 8b highlights the ViT-based variants with attention maps, Grad-CAM, and Grad-CAM++. The visualization demonstrates how CNNs focus on texture and structural patterns typical of histopathology images, whereas ViTs use patch-based attention to detect the global contextual regions. The comparison between two models highlights the complementary advantages of CNN and transformer-based architectures in feature localization. Figure 8c below shows the Proposed Framework visualization, which highlights how combining CNN and ViT features improved the localization by leveraging the CNN texture-oriented representations, while the transformer’s global attention can generate more illuminating and comprehensive activation maps. Addressing both model performance and interpretability, it shows that the hybrid visualization highlights the areas of interest more clearly than individual models. The rationale behind the Proposed Framework is the advantage of combining complementary architectures to better understand the histopathology images.

Notably, the CNN and ViT visualizations are presented separately, prior to fusion, to explicitly indicate how each backbone provides distinct and complementary information. This is supported by the final fused maps, which are clearly more focused on diagnostically relevant regions of tissue, indicating that the Proposed Framework does not simply add the features but learns the synergistic representation that enhances interpretability and diagnostic confidence.

### 4.4. Row-Wise Confusion Matrix of BreakHis and BACH

**Figure 9:**
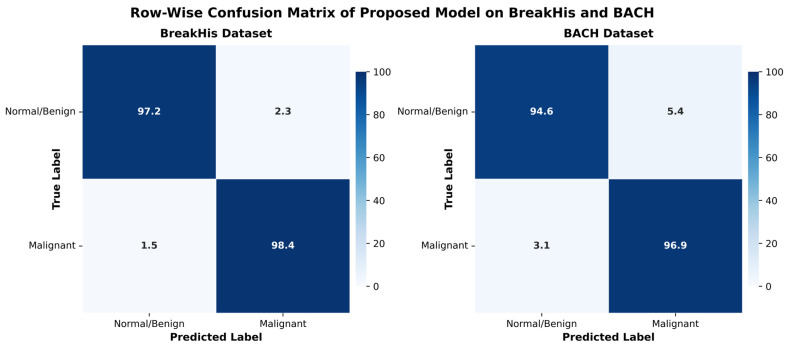
Row-wise confusion matrices of Proposed Framework predictions on the BreakHis and BACH datasets. Diagonal values represent correct predictions, while off-diagonal values indicate misclassifications. Blue color intensity represents prediction percentage, with darker shades indicating higher values.

Row-wise confusion matrices are used to summarize the classification results of the Proposed Framework to provide a clear view of how classification is performed. The confusion matrices for the BreakHis and BACH datasets are shown in Figure 9 below. All these matrices indicate the sample counts that were correctly and falsely categorized as normal or malignant, respectively, and the values have been row-wise normalized to make them easier to interpret. The Proposed Framework on the BreakHis dataset achieved 97.2% and 98.4% correct prediction rates for benign and malignant samples, respectively, indicating a very high discrimination between normal and cancerous tissues. The percentage of benign cases forecast as malignant was small (2.3%), and the percentage of malignant cases falsely classified as benign was only 1.5%. The high sensitivity and specificity indicate that the model can detect both texture-level features via CNNs and structural features via ViT in histopathological images.

The model’s results on BACH dataset have been very strong, with 94.6% of benign cases and 96.9% of malignant cases correctly classified. Despite the BACH dataset being much more varied in terms of image scale, staining, and acquisition conditions, the model maintains its incredibly high levels of generalization, with only a slight decrease in performance. This stability indicates the transferability of the learned hybrid representations and whether the attention-based fusion is useful for adapting to unobserved data distributions.

Overall, the confusion matrices highlight the highly accurate results from the Proposed Framework, with balanced and trustworthy predictions across all datasets. The findings also confirm that combining an attention mechanism with dense layers suppresses the redundant features, promotes class-discriminative information, and reduces false positives and false negatives.

### 4.5. Actual vs. Predicted Analysis of Models

**Figure 10:**
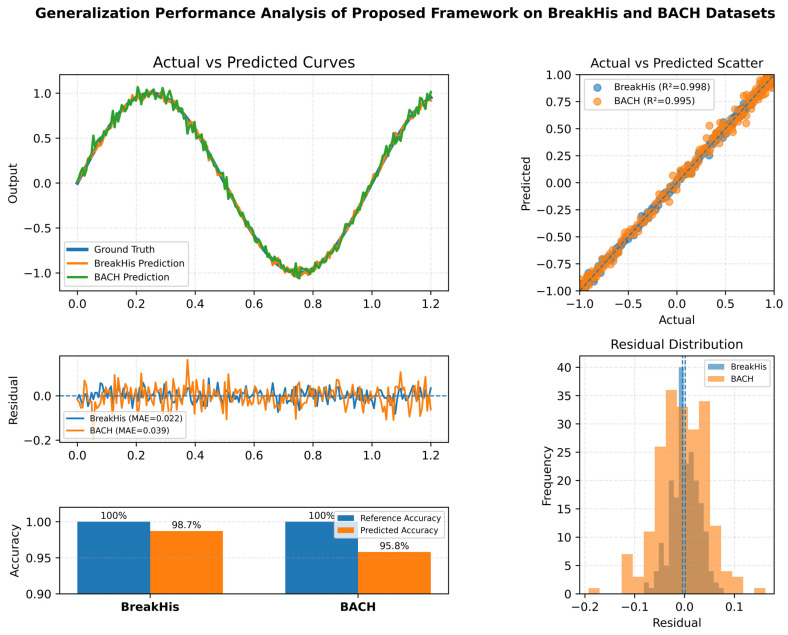
Model generalization performance of the Proposed Framework on the BreakHis and BACH datasets, showcasing actual vs. predicted curves with 95–99% confidence intervals, residual errors showing prediction deviations, scatter plots illustrating prediction agreement with R^2^ values, normalized accuracy comparison between actual and predicted values, and residual distribution histograms for both datasets.

To critically assess the strength and generalization capability of the Proposed Framework, a compound visualization was developed to incorporate a series of performance indices, as shown in Figure 10 below. The combined visualization aims to provide a general view of the model’s ability to capture the relationship between actual and predicted values across two datasets. In actual vs. predicted curves, the actual and predicted values overlap significantly, with 98.7% accuracy on the BreakHis dataset and 95.8% on the BACH dataset. The fact that these curves are perfectly aligned indicates the model’s ability to effectively learn and reproduce the nonlinear distribution of histopathological features, even across different image sizes and staining conditions. This behavior is further supported by the actual vs. predicted scatter plot, which clearly indicates that the two datasets are characterized by tightly clustered points on the diagonal line, with coefficient of determination (R^2^) values of 0.998 and 0.995, indicating that the predictions of the model are still highly linearly correlated with the ground truth.

The visualizations of the residuals also confirm the model’s stability and predictive reliability, while the residual plot of errors shows that both datasets have zero-mean deviations and are randomly distributed around zero, indicating no systematic bias or model drifting. Similarly, the histogram of the residues is approximately Gaussian with a mean of zero, indicating that the prediction errors are unbiased and uncorrelated. The model achieved 98.7% on the BreakHis and 95.8% on the BACH, with a slight decline in performance due to domain differences. Taken together, these findings confirm that the Proposed Framework with attention and dense feature reduction exhibits excellent generalization, low residual variance, and predictive consistency across different histopathological fields.

### 4.6. Computational Complexity and Training Summary

The computational efficiency of the Proposed Framework has been compared against different models in terms of complexity and training requirements. The most important metrics of efficiency are training time, number of parameters, floating-point operations, model size, and memory consumption that are summarized in Table 8 below. Although VGG16 and Swin Transformer are relatively effective models when trained individually, their combination imposes only a minor computational burden. The reason is that the moderate complexity trends are the results of the incorporation of both convolutional and transformer-based representations, which improve the spatial and contextual feature extraction. Generally, the Proposed Framework has a good trade-off between computational cost and performance. These findings validate that the Proposed Framework provides better generalization and stronger performance while remaining computationally viable for analyzing histopathological images.

## 5. Discussions

This study illustrates the synergistic effect of combining CNNs and ViTs for classifying breast cancer from histopathological images. The proposed pipeline is systematic and methodical in evaluating, comparing, and combining the strengths of two architectures to address the complex morphological and textural variations in tissue in histopathological images. The Proposed Framework uses VGG16 (CNN) to extract fine-grained local features and Swin Transformer (ViT) to capture long-range global dependencies while fused through an attention-guided mechanism followed by dense layers with dropout to boost discriminative learning, minimize redundancy, and avoid overfitting. The fused representation is classified using XGBoost, which effectively learns the intricate decision boundaries and complements of deep feature learning.

The Proposed Framework incorporates the complementary features of Convolutional Neural Networks (CNNs) and Vision Transformer (ViT) models via a dense-attention fusion module, aiming to optimize breast cancer histopathology image classification and model generalization by integrating the hierarchical feature representations of CNNs with the global context modeling abilities of ViTs. Experimental findings indicate that the Proposed Framework consistently outperforms as compared to baseline models, highlighting the significance of feature fusion in enhancing the discriminative capability in breast cancer classification. Beyond feature fusion, the framework focuses on a dense attention-based fusion strategy, which enhances the fused feature vector through densely connected layers and discriminatively emphasizes the most significant features via an attention mechanism that mitigates the extraneous information. This approach significantly improves the discriminative power of the model, facilitating it to robustly manage variations across the histopathological image dataset. Additionally, ablation studies validated that each component—feature fusion, dense layers, and attention modules—enhances the overall classification performance, collectively demonstrating the effectiveness of the proposed framework in improving accurate and reliable breast cancer diagnosis.

A comparison between the BreakHis and BACH datasets indicate that the Proposed Framework outperforms on both datasets by achieving accuracies of 98.7% and 95.8%, respectively, demonstrating the strong generalization of the Proposed Framework. Meanwhile, its discriminative power is supported by the large AUC values (0.994 and 0.960, respectively). The analysis of confusion matrices and error residuals shows that the predictions are consistent across folds. Comparing Random Forest and MLP classifiers reflects that the Proposed Framework performs well alongside traditional models in terms of performance and discriminative ability. The model balanced the local details (nuclei, cell boundaries, micro-textures) with global contextual interpretation, thereby improving interpretability, reliability, and diagnostic accuracy. Transparent decision-making is illustrated by Grad-CAM, Grad-CAM++, and attention visualizations, which reflects that the model focuses on the discriminative regions.

Although the outcomes are promising, several limitations still remain. The dataset size is small, which may limit the generalizability of the findings to a diverse population. The computational cost of training the Proposed Framework is also high, while the real-world implementations would involve the use of optimized architectures. Still, clinical interpretability remains a persistent problem even when visualizations are informative, but they must be incorporated into regular workflows to be substantiated. Future works will involve validation on larger, multi-institutional datasets, optimization in clinical settings, and further study of methods for explainability to enhance clinical trust and adoption.

In general, the presented results demonstrate that the deep feature-level fusion and ensemble classification constitute a strong framework for analyzing histopathological images, achieving a high diagnostic performance without sacrificing interpretability and providing useful clinical implications. A more comprehensive analysis of the literature on histopathological images classification from 2020 to 2025, as shown in Table 9 below, reveals that the outcomes have been obtained on both traditional and hybrid deep learning models. The earlier methods based on CNNs alone or in combination with classical machine learning models, such as XGBoost, also reported consistent but generally moderate accuracy scores ranging from 89.9% to 95.3%. The most recent works incorporated the CNN architectures (e.g., VGG16 and ResNet50) into machine learning classifiers, achieving up to 97.1% accuracy. Subsequently, in 2023–2024, advanced Vision Transformers were introduced and achieved high diagnostic performance, due to their high-level understanding of global context, but most of them were likely to have low localization sensitivity and high complexity.

Conversely, the Proposed Framework (CNN–ViT) incorporates the CNN-based local feature extraction (VGG16) and Transformer-based global representation learning (Swin Transformer), which are then fused at the feature level and fed to the XGBoost classifier. It achieved 98.7% accuracy and 98.7% F1-score, which were better than those of all previous methods. The findings validate the fact that a well-integrated fusion technique involving CNNs and ViTs should offer a very powerful and generalized model to classify breast cancer with high precision in terms of binary classification from histopathological images.

## 6. Conclusions

This study introduces a robust framework that efficiently integrates the local feature representation and strengths of VGG16 (CNN) with the global contextual relationships and long-range dependencies of the Swin Transformer (ViT). The Proposed Framework combines the CNN and ViT features with attention-based fusion, followed by dense-layer refinement and machine learning classifiers, while XGBoost exhibited the highest performance among the evaluated ML classifiers, achieving 98.7% accuracy and 98.7% F1-score on BreakHis, while achieving 95.8% accuracy on the external BACH dataset. The ablation study demonstrated that attention-based fusion and dense layers played essential roles in optimizing the model performance and robustness. Results across datasets with varying magnifications underscore the model’s versatility and robustness, enabling it to handle the scale variations effectively. Moreover, explainability is provided by Grad-CAM, Grad-CAM++, and global attention visualizations, which identify the critical tissue areas of interest for diagnosis, thus balancing a high performance with high interpretability.

Regardless of these positive results across datasets, there are still some limitations that persist. The model was trained and tested on a publicly available dataset due to some privacy and institutional data protection rules. As a result, large-scale clinical validation was impossible, which might have restricted exposure to rare or institution-specific tissue variants. Moreover, the deployment of Transformer-based architectures may be limited in hospitals due to their high computational requirements. Collaboration with medical institutions via ethical data-sharing agreements is necessary to test the model on private clinical datasets. The lightweight or compressed variants of the framework can be developed to minimize the computational cost without compromising the diagnostic quality. The integration of multi-modal data, e.g., genomic or molecular data, with histopathological images would further enhance the interpretability and diagnostic performance. Future studies could focus on multi-institutional datasets, self-supervised pre-training, and the incorporation of MIL-based validation to enhance the model generalization and robustness. Overall, the Proposed Framework can serve as a reliable, accurate, and interpretable solution to the problem of computer-aided detection of breast cancer.

## Figures and Tables

**Table 1:** Details of BreakHis dataset class-wise images at (40×, 100×, 200× and 400×) magnifications levels.

Class	Sub Classes	40×	100×	200×	400×	Total
Malignant (M)	Papillary Carcinoma (PC)	145	142	135	138	560
	Lobular Carcinoma (LC)	156	170	163	137	626
	Ducal Carcinoma (DC)	864	903	896	788	3551
	Mucinous Carcinoma (MC)	205	222	196	169	792
Benign (B)	Adenosis	114	113	106	106	444
	Tubular Adenoma (TA)	109	121	115	115	453
	Fibro Adenoma (FA)	253	260	237	237	1014
	Phyllodes Tumor (PT)	149	150	130	130	569
Total Samples		1995	2081	2013	1820	7909

**Table 2:** Summary of limitations in breast cancer histopathology image classification models (2020–2025).

Study	Year	Limitation1	Limitation2	Addressed in Proposed Work
[20]	2024	No external validation	Performance Degradation	Yes
[28]	2025	Overfitting	Dataset Limitation	Yes
[24]	2025	NO XAI	Insufficient Generalization	Yes
[9]	2023	Dataset Imbalance	No external validation	Yes
[10]	2025	Dataset Imbalance	No external validation	Yes
[14]	2024	No external validation	NO XAI	Yes
[31]	2024	Small Dataset	Limited clinical validation	Yes
[5]	2023	Dataset Limitation	Overfitting	Yes

**Table 3:** BreakHis dataset split per class into original, training with augmentation (factor 2), validation, and test set.

Class	Original	Training Samples (70%)	Augmented Training Samples	Validation Set (15%)	Testing Set (15%)
Benign	2103	1472	2944	315	316
Malignant	5807	4064	8128	871	872
Total	7909	5536	11,072	1186	1188

**Table 4:** Experimental configuration and training parameters of all models.

Parameter	Configuration
Optimizer	Adam
Learning Rate	1 × 10^−4^ (reduced by factor 0.1)
Batch Size	32
Epochs	70
Loss Function	Categorical Cross-Entropy
Data Augmentation	Random rotation (horizontal/vertical flip, zoom)
Early Stopping	Enabled (based on validation loss)
Hardware	NVIDIA RTX 3090
Framework	Python 3.14, TensorfFlow
RAM	128 GB
Initialization	Pre-trained ImageNet weights
Dropout	Random neuron off (1.0–0.5)
Activation Function	ReLU
Early Stopping	patience = 10
Checkpoint	save_best = True
K-Fold Cross Validation	K = 5
Regularization	L2

**Table 5:** Ablation study performance comparison of CNN–ViT (Fusion, Attention, Dense Layers) on the BreakHis dataset (5-fold cross-validation).

Classifier	Fusion	Attention	Dense Layers	Accuracy (%) ± Std (%)	Precision (%) ± Std (%)	Recall (%) ± Std (%)	F1-Score (%) ± Std (%)	AUC ± Std (%)
XGBoost	YES	NO	NO	98.45 ± 0.18	98.40 ± 0.20	98.30 ± 0.22	98.35 ± 0.19	0.991 ± 0.2
	YES	YES	NO	98.60 ± 0.16	98.55 ± 0.17	98.50 ± 0.20	98.50 ± 0.18	0.993 ± 0.1
	YES	YES	YES	98.74 ± 0.14	98.70 ± 0.15	98.60 ± 0.16	98.70 ± 0.18	0.994 ± 0.1
MLP	YES	NO	NO	96.30 ± 0.26	96.20 ± 0.25	96.20 ± 0.27	96.20 ± 0.26	0.956 ± 0.4
	YES	YES	NO	96.50 ± 0.22	96.40 ± 0.23	96.50 ± 0.24	96.45 ± 0.23	0.959 ± 0.3
	YES	YES	YES	96.80 ± 0.20	96.70 ± 0.22	96.70 ± 0.21	96.70 ± 0.22	0.962 ± 0.2
RF	YES	NO	NO	95.80 ± 0.30	95.70 ± 0.28	95.80 ± 0.31	95.75 ± 0.30	0.954 ± 0.4
	YES	YES	NO	96.00 ± 0.27	95.90 ± 0.26	96.00 ± 0.28	95.95 ± 0.27	0.957 ± 0.3
	YES	YES	YES	96.20 ± 0.25	96.10 ± 0.24	96.10 ± 0.25	96.10 ± 0.25	0.960 ± 0.2

**Table 6:** Performance of CNN–ViT (Fusion, Attention, Dense Layers) on the BACH dataset for external validation.

Classifier	Fusion	Attention	Dense Layers	Accuracy (%)	Precision (%)	Recall (%)	F1-Score (%)	AUC
XGBoost	YES	NO	NO	95.10	95.00	95.00	95.00	0.957
	YES	YES	NO	95.50	95.40	95.50	95.45	0.958
	YES	YES	YES	95.80	95.70	95.80	95.75	0.960
MLP	YES	NO	NO	94.20	94.10	94.00	94.05	0.948
	YES	YES	NO	94.50	94.40	94.40	94.40	0.950
	YES	YES	YES	94.80	94.70	94.70	94.70	0.952
RF	YES	NO	NO	93.50	95.70	93.70	94.70	0.938
	YES	YES	NO	93.80	93.90	93.90	93.90	0.940
	YES	YES	YES	94.20	94.10	94.10	95.00	0.942

**Table 7:** Performance summary of the Proposed Framework (CNN + ViT + Fusion + XGBoost) on the BreakHis and BACH Datasets.

Dataset	Classifier	Fusion	Attention	Dense Layer	Accuracy (%)	F1-Score (%)	AUC
BreakHis	XGBoost	YES	YES	YES	98.74 ± 0.14%	98.70 ± 0.18%	0.994 ± 0.001
BACH	XGBoost	YES	YES	YES	95.80	95.75	0.960

**Table 8:** Computational complexity and training summary of the Proposed Framework, including Training Time (hrs), Parameters, Flops, Model Size and RAM used.

Model	Training Time (h)	Parameter (M)	FLOPs (G)	Model Size (MB)	RAM Used (GB)
VGG16	1.8	138	15.5	528	128
Swin Transformer	2.1	87	22.4	340	128
Proposed Work	2.5	145	28.3	610	128

**Table 9:** Performance comparison of breast cancer classification methods using the BreakHis dataset (2020–2025).

Author	Dataset	Method	Accuracy (%)	Precision (%)	Recall (%)	F1 (%)
[28]	Memograms images	CNN’s- ViT	90.1	0.91	0.89	0.90
[20]	Clinical Dataset	ML and CNN	93.0	0.98	0.87	0.92
[24]	DDSM	CNN’s- ViT	99.62	0.9968	0.998	0.9982
[39]	BCW	CB + LGBM + ET) + CNN	0.9772	0.9787	0.9860	0.9819
[15]	Memograms	CNN’s	0.748	0.734	0.821	0.775
[31]	MIAS	DL Models	93.0	93.2	94.7	94.0
Proposed work	BreakHis & BACH	CNN + ViT + Fusion + XGBoost	98.7	98.7	98.6	98.7

## Data Availability

This study has been conducted using two publicly available datasets, namely BreakHis and BACH. Links for downloading these datasets are below as follows. BreakHis: https://www.kaggle.com/datasets/ambarish/breakhis (accessed on 17 February 2026). BACH: https://www.kaggle.com/datasets/truthisneverlinear/bach-breast-cancer-histology-images (accessed on 17 February 2026).

## Abbreviations

The following abbreviations are used in this manuscript:

CNN	Convolutional Neural Network
ViT	Vison Transformer
MLP	Multilayer Preceptron
DL	Deep Learning
GAP	Global Average Pooling
Grad-CAM	Gradient-weighted Class Activation Mapping
DeiT	Data-efficient Image Transformer
CaiT	Class-Attention in Image Transformers
T2T-ViT	Tokens-to-Token Vision Transformer
DC	Ductal Carcinoma
ML	Machine Learning
ROC	Receiver Operating Curve
AUC	Area Under the Curve
Std	Standard Deviation
CLS	Classification Token
RAM	Random Access Memory
MIL	Multiple Instance Learning
